# Exchange transfusion combined with artesunate (ET-AS) as a safe and effective therapy in severe *P. falciparum* malaria: a case series

**DOI:** 10.1186/s12879-024-09381-2

**Published:** 2024-06-19

**Authors:** Jingjing Zhang, Lulu Chen, Min Zhang, Mingkang Yao, Saisai Ren, Haihui Liu, Yanan Min, Yan Jia, Yanling Tao, Hao Zhang

**Affiliations:** 1 Department of Hematology, Affiliated Hospital of Jining Medical University, Jining, 272029 Shandong Province China; 2 Institute of Blood and Marrow Transplantation, Jining Medical University, Jining, Shandong Province China; 3 The First School of Clinical Medicine, Fujian Medical University, Fuzhou, Fujian Province China; 4 Department of Pediatric Hematology, Affiliated Hospital of Jining Medical University, Jining, 272029 Shandong Province China

**Keywords:** Exchange Transfusion, Artesunate, Severe *P. falciparum* malaria, Treatment, Case series

## Abstract

the mortality associated with severe malaria due to *Plasmodiun* falciparum remains high despite improvements in malaria management. Case prensentation: this case series aims to describe the efficacy and safety of the exchange transfusion combined with artesunate (ET-AS) regimen in severe *P. falciparum* malaria. Eight patients diagnosed with severe *P. falciparum* malaria were included. All patients underwent ET using the COBE Spectra system. The aimed for a post-exchange hematocrit of 30%. Half the estimated blood volume was removed and replaced using fresh frozen plasma. The regimen was well-tolerated without complications. The parasite clearance time ranged from 1 ~ 5 days. Five patients with cerebral malaria exhibited full improved consciousness within 3 days, while patient2 with hemolysis improved on day 2. Liver function improved within 1 ~ 6 days, and patient 1 and patient 6 showed improvements renal function on days 18 and 19, respectively. The length of intensive care unit stay range from 2 ~ 10 days, and all patients treated with ET-AS remained in the hospital for 3 ~ 19 days. Conclusions: these preliminary results suggest that ET-AS regimens are a safe and effective therapy for severe *P. falciparum* malaria and can benefit patients in clinical settings.

**Supplementary Information:**

The online version contains supplementary material available at 10.1186/s12879-024-09381-2.

## Background

Severe malaria, caused by *P. falciparum*, is the primary cause of death from malaria [1]. Even after immediate parenteral therapy with artesunate (AS), the mortality rate remains at 15 to 25% [2]. Currently, no effective therapeutic agents are available for reducing the mortality of severe *P. falciparum* malaria [1]. Thus, further thorough explorations of adjuvant therapies associated with antimalarial drugs are eagerly needed.

Red cell exchange (RCE) is the removal of abnormal red blood cells from the patient’s blood and replacing them with normal donor red blood cells using a cell separator [3]. RCE is said to reduce parasite load, remove toxic substances and reduce microcirculatory sludging, which offers adjunct and rapid approach in severe *P. falciparum* malaria [4]. Some researchers have suggested therapeutic benefit of RCE for patients with pulmonary, renal, and cerebral complications in severe *P. falciparum* malaria [5, 6]. Unfortunately, no large-scale randomized clinical trials have been conducted to date comparing RCE as an adjunct therapy for malaria [7, 8]. This type of treatment as an adjunct therapy for severe *P. falciparum* remains controversial [9].

Therapeutic plasma exchange (TPE) is a procedure that involves the removal of pathogenic substances, metabolites, and toxins from the plasma of patients with various medical conditions and replacing them with normal donor plasma or albumin [10, 11]. In recent years, TPE has been widely used in clinical practice, which can effectively reduce the mortality of sickle cell disease, thrombotic thrombocytopenic purpura [12]. In the 1980s, TPE was proposed as a means of treating severe malaria [13]. However, no evidence exists to support its efficacy in reducing the mortality of severe malaria.

The recommendation of using exchange transfusion (ET) as adjuvant therapy for severe plasmodium falciparum (*P. falciparum*) malaria remains controversial. The studies reported so far lack statistical power, comparability of control groups, and non-standardized treatment regimens [14–16]. Only a single inadequately powered randomized control trail found that ET does not appear to provide significant benefit [17]. Several lines of pathophysiology evidence support the efficacy of ET in the treatment of severe *P. falciparum* malaria [14]. ET can not only reduce the infected blood cells but also reduce inflammatory cytokines such as tumor necrosis factor (TNF)-α [18, 19]. Sporadic cases have demonstrated that the administration of adjunct ET can improve the clinical symptoms of patients when antimalaria drugs have failed in clinical practice for severe *P. falciparum* malaria [15, 20, 21].

Artesunate is the first-line antimalarial drug for the treatment of severe malaria [22]. But antimalarial therapy alone cannot improve pathological damage [1]. Considering ET efficacy in reducing infected red blood cells and plasma (including parasitic antigens and toxic products), patients with severe malaria with organ dysfunction represent prime candidates for ET-AS [23, 24].

Herein, we present eight adult patients with severe *P. falciparum* malaria who were successfully salvaged with ET-AS. Table 1 provides a summary of clinical characteristics and ET procedure dates for these eight patients. The combination therapy was well tolerated. The outcomes of the eight patients are summarized in Table 2. These preliminary results suggest that ET-AS regimens could be beneficial patients with severe *P. falciparum* malaria.

**Table 1:** Patient characteristics and ET procedural data

characteristics	Case 1	Case 2	Case 3	Case 4	Case 5	Case 6	Case 7	Case 8
Age/Gender	54/Male	39/Male	38/Male	41/Male	49/Male	41/Male	41/Male	27/Male
Weight (Kg)	89	80	80	71	80	80	77	65
Time from return and onset of symptoms (days)	7	21	15	18	12	5	35	1
Falciparum (%)	18	15	15	12	20	25	15	32
HB (g/L)	119	48	109	58	79	119	124	118
HCT%	33.7	14.1	31.5	18.7	23.8	32	35.3	33.4
PLT (×109)	127	100	9	75	17	18	86	60
Ret (%)	0.93	6.71	0.70	0.56	2.44	0.96	2.89	0.42
Cr (umol/L)	812.8	-	-	-	-	635.4	-	-
GCS	-	5	-	-	3	3	3	3
BP (mmHg)	-	-	-	-	-	-	-	88/59
AST (U/L)	43	118	101	-	-	-	-	-
ALT (U/L)	-	-	105.7	-	-	-	-	-
Total Bilirubin (umol/L)	44	42.5	42	-	65	339	37.8	-
Treatment (before admission)	AS	AS	-	-	AS	Artemisia	-	AS
ET	3	3	3	3	4	6	3	3
Red cell (U)	8	8	8	8	11	10	12	7
Plasma (ml)	3000	2500	2500	2000	2500	2500	2500	1000

Abbreviations: HB, hemoglobin; HCT, hematokrit; PLT, platelet; Ret, reticulocyte; Cr, creatinine; GCS, Glasgow Coma Scale score; BP, blood pressure; AST, aspartic transaminase; ALT, glutamic-pyruvic transaminase; TBIL, total bilirubin. ET, exchange transfusion;“-”means laboratory test results normal

**Table 2:** Outcomes

	Case 1	Case 2	Case 3	Case 4	Case 5	Case 6	Case 7	Case 8
Time of parasite clearance (days)	2	2	1	2	5	3	3	1
Falciparum (%)	0	0	0	0	0	0	0	0
Began time for GCS improvement (hours)	none	18	none	none	12	8	24	4
Time for hemolysis improvement (days)	none	2	none	none	none	none	none	none
Time for liver function improvement (days)	5	none	4	none	5	5	4	none
Time for renal function improvement (days)	18	none	none	none	none	19	none	none
Length of ICU(days)	none	5	none	none	3	10	2	2
Remained in hospital (days)	18	6	6	4	7	19	4	3

Abbreviations: GCS, Glasgow Coma Scale score;

## Case presentation

### Case 1

A 54-year-old male was admitted with a history of fever, diarrhea, vomiting, and decreased urine output for the past 5 days. These symptoms started after returning from Africa 7 days ago. During the physical examination, a palpable spleen 2 cm below the left costal margin was identified.

Laboratory data showed a hemoglobin (HB) level of 119 g/L, hematocrit (HCT) of 33.7%, and a platelet count (PLT) of 127 × 10^9^/L. The initial peripheral blood smear showed the presence of 18% *P. falciparum* parasites. Lactic dehydrogenase (LDH) was 330U/L, creatinine (Cr) 812.8umol/L, Aspartate transaminase (AST) 43U/L, total bilirubin (TB) 44.0umol/L. The patient had received treatment with artesunate before admission, but had not showed any improvement in fever or other clinical symptoms. Moreover, the patient was found to have hyperparasitemia, which prompted immediate ET. The patient’s Cr levels were excessively high, necessitating intubation and renal replacement therapy. The patient’s fever subsided and renal function improved by day 3. On admission on day 18, a review Cr, urine volume, and laboratory data showed normal levels. The patient was discharged on day 18 to complete oral anti-malarial treatment.

### Case 2

A 39-year-old male was referred to the intensive care unit (ICU) after experiencing a fever, chills, cough, and yellow phlegm for 7 days. These symptoms started 21 days ago, shortly after his return from Africa. Upon presentation, the patient exhibited a poor level of consciousness and was uncooperative during the physical examination. The Glasgow Coma Scale (GCS) score upon arrival was 5/15.

Laboratory results revealed HB level of 48 g/L, HCT of 14.1%, and PLT of 100 × 10^9^/L, Reticulocyte 6.71%. The initial peripheral blood smear showed the presence of 15% *P. falciparum* parasites. Additionally, Cr was measured at 167.9umol/L, random blood glucose at 11.42mmol/L, ketone body at 4.1 mg/ml, serum cholinesterase at 2012U/L, TB at 42.5umol/L. Despite having received a single intravenous artesunate treatment at another hospital, the patient’s level of consciousness continued to deteriorate. As a result, ET treatment was administered. Within 18 h of receiving ET, the patient mental status showed significant improvement. On day 4, the patient’s fever subsided, and he regained a normal mental status with a GCS score of 15/15. Subsequently, the patient was discharged on day 6 with instructions to complete an oral anti-malarial treatment.

### Case 3

A 38-year-old male was referred to the emergency room due to a 10-day history of fever, weak, and poor appetite after returning to China from African 15 days prior. During the physical examination, a palpable spleen 3 cm below the left costal margin was identified.

Laboratory results revealed an HB level of 109 g/L, HCT of 31.5%, and PLT of 9 × 10^9^/L. The initial peripheral blood smear showed the presence of 15% *P. falciparum* parasites in the patient blood. LDH levels were found to be 624U/L, TB at 42umol/L, AST at 101U/L, ALT at 105.7U/L. After admission, the patient was diagnosed with hyperparasitemia and liver function damage, and immediate ET treatment was administered. On day 4, the PLT returned to 50 × 109/L, and the liver function returned to normal on day 2. The patient was discharged on hospital day 6 to complete oral anti-malarial treatment.

### Case 4

A 41-year-old patient presented to our hospital with persistent fever for 18 days, fatigue and poor appetite after returning from Africa 18 days before. During the physical examination, distended abdomen and palpable splenomegaly were observed.

Laboratory results revealed an HB level of 58 g/L, HCT of 18.5%, and PLT of 75 × 10^9^/L. The initial peripheral blood smear showed the presence of 12% *P. falciparum* parasites in the patient’s blood. After admission, the patient was diagnosed with hyperparasitemia and immediately received ET treatment. The time to parasite clearance in the patient was 3 days. Subsequently, the patient was discharged on hospital day 4 to complete oral anti-malarial treatment.

### Case 5

A 49-year-old male presented to our ICU with an 8-day history of fever. He had returned from Angola 12 days prior to his presentation and had not taken any malaria prophylaxis. On admission, the patient exhibited a high temperature of 39.9℃ and a poor level of consciousness. He was uncooperative during the physical examination, and his GCS score upon arrival was 3/15.

Laboratory results revealed an HB level of 79 g/L, HCT of 23.8%, and PLT of 17 × 10^9^/L. The initial peripheral blood smear showed the presence of 20% *P. falciparum* parasites. TB measure at 65.0umol/L. Immediate ET treatment was administered upon admission. Within 12 h of receiving ET, the patient mental status showed significant improvement, and by day 2 they had fully recovered. And the patient was discharged from ICU on day 3. Complete parasite clearance was achieved on day 5, and the patient was subsequently discharged on day 7 to complete oral anti-malarial treatment.

### Case 6

A 41-year-old man presented to a local hospital with a 5-day history of fever, diarrhea and headaches following he return from Congo. He was diagnosed with malaria and was given intravenous Artemisia. However, on the fourth day, the patient fell into a coma and experienced oliguria. He was then transferred to our ICU. Upon arrival, he was unresponsive during the physical examination, and his GCS was 3/15.

Laboratory results revealed an HB level of 119 g/L, HCT of 32%, and PLT of 18 × 10^9^/L. The initial peripheral blood smear showed the presence of 25% *P. falciparum* parasites. His Cr level was measure at 635.4umol/L, and TB was measure at 339.0umol/L. Immediate ET therapy was administered. Eight hours after the first cycle of ET, the man emerged from coma. The patient’s mental status recovered on day 3, and the GCS improved to 15/15. However, on the third day of treatment, the patient’s Cr levels continued to rise. He was intubated and treated with continuous renal replacement therapy. Symptoms of oliguria persisted until day 11, but the Cr level remained high at 800.6umol/L, and the patient required dialysis on day 18. On admission on day 19, a review of PLT, TB, and Cr showed normal levels, and the patient was discharged on Day 19 to complete oral anti-malarial treatment.

### Case 7

A 41-year-old male was transferred to our hospital with 5-day history of fever, chills, joint pain, and diarrhea after returning from Africa 35 days prior. He had lost consciousness for three hours before admission to local history. Upon admission to our ICU, the patient was unresponsive during physical examinations and his GCS was 3/15.

Laboratory results revealed an HB level of 124 g/L, HCT of 35.3%, and PLT of 86 × 109/L. The initial peripheral blood smear showed the presence of 15% *P. falciparum parasites.* His TB was measure at 37.8umol/L. Immediate ET therapy was administered. After approximately 24 h following the first ET, the patient regained consciousness. On the third day of admission, his GCS upon arrival was 15/15. By the 4th day, his physical condition had improved to normal, and the patient was discharged to complete oral anti-malarial treatment.

### Case 8

A 27-year-old male was admitted to the local hospital with a 4-day history of febrile, chills, and headache. One day before his admission, he suddenly turned unconscious accompanied by recurrent febrile seizures. The local hospital considered the diagnosis of “malaria”, and gave him intravenous artesunate 120 mg. Due to the continuing deterioration, he was transferred to our hospital. On admission, the patient exhibited hypotension of 88/59mmHg and a poor level of consciousness. He was uncooperative during the physical examination, and his GCS score upon arrival was 3/15.

Laboratory results revealed an HB level of 118 g/L, HCT of 33.4%, and PLT of 60 × 109/L. The initial peripheral blood smear showed the presence of 32% *P. falciparum parasites.* As the patient’s level of consciousness continued to deteriorate, immediate ET therapy was administered. In order to address the patient’s hypotension, intravenous access was established and adequate fluids were given. Noradrenaline was used during the exchange transfusion procedure. Three hours after the first cycle of ET, the man emerged from coma and were able to answer some simple questions. On the third day of admission, his GCS on arrival was 15/15. On the fourth day, a review of PLT and peripheral blood smear showed normal levels. The man was then discharged to complete oral anti-malarial treatment.

#### Basic treatment

The patients were given AS in a dose of 2.4 mg/kg on 0, 12, and 24 h(h)and then daily after that until the initiation of oral therapy [25]. Continuous renal replacement therapy was performed in patients with acute renal failure. Apheresis platelets and packed cells were administered to patients with a platelet count < 20 × 10^9^/L and Hb <60 × 10^9^/L. Antibiotics were initiated when bacterial superinfection was suspected.

#### Exchange transfusion

ET was performed using a COBE Spectra Blood Cell Separator (Terumo BCT, Lakewood, Co, USA) for the patients who met one of the following criteria: parasitemia of more than 10% infected RBCs, serum creatinine>250umol/L; systolic pressure < 80mmHg with cold extremities; Glasgow Coma Scale of less than 12; total serum bilirubin > 50µmol/L [26]. Patients received ET every 24 h. A double-lumen central venous catheter was inserted in the right antecubital vein, which was used to transfuse RBCs and plasma (the ‘in-flow’ pathway). Another catheter was inserted into the antecubital vein of the opposite arm, which was used to collect blood into a donor set (the ‘out-flow’ pathway). Acid citrate dextrose-A (ACD-A) was used as the anticoagulant. The anticoagulant ratio was 1:14. All patients were administered 10 ml of 10% calcium gluconate to prevent hypocalcemia. The RCE program replaced infected red cells, with pre-and desired post-exchange hematocrit being entered. The aimed for a post-exchange hematocrit of 30%. For TPE, we removed half the estimated blood volume from each patient, and 100% replacement was provided using fresh frozen plasma. For patient 8, who had hypotension, reduced blood volume was removed. When the parasite count had dropped below the level of 1% infected RBCs, the ET was ceased. Blood pressure, pulse rate, oxygen saturation, and urine output were monitored during the ET procedure.

## Discussion and conclusions

Severe malaria is caused by *P. falciparum* malaria with high mortality rates [27]. In our case series, eight patients with severe *P. falciparum* malaria were treated with an ET-AS regimen. There was no severe adverse or death in all cases. The use of ET for treatment of severe malaria is controversial. It is not recommended by the WHO guidelines as it requires a relatively large volume of blood and intensive nursing care thereby carries significant risks. Meanwhile, the lack of consensus on the indications, benefits and dangers involved or on practical details (e.g. the volume of blood) makes the conduction of ET in malaria treatment quite variable and highly experience-demanded.

In our case series, we observed a parasite clearance time ranging from 1 to 5 days, which is comparable to the findings of the Tropnet Severe Malaria Study [28]. According to their research, patients with parasitemia levels of 5% or higher showed a parasite clearance time of 60 to 92 h. And in another trial reported by Kreeft Meijer Vegter et al., they found that ET could not significantly contribute to parasite clearance in artesunate-treated individuals [9]. Considering the limited sample sized of this study, the efficacy of ET-AS in parasite clearance should be verified by further study.

The clinical picture of severe malaria may persist or even worsen despite parasite clearance from blood [29]. According to our results, the ET-AS regimen rapidly improves cerebral malaria, liver function, hemolysis and blood routine examination. This case series result is consistent with the findings of the previous study [9, 30]. The length of ICU stay range from 2 ~ 10 days, and all patients treated with ET-AS remained in the hospital for 3 ~ 19 days, significantly shorter than the findings reported by previous studies [30, 31]. Malaria-associated acute kidney injury with severe malaria is associated with increased mortality [32]. Renal replacement therapy has been found to be associated with improved survival rate and recovery of renal function [33]. Herein, patients1 and 6, who demonstrated severe complications of renal dysfunction (failure), were applied with hemodialysis along with the ET-AS treatment on the first day of admission.

Based on the present findings, the main arguments in favor of the superior efficiency of ET-AS include the advantageous outcomes associated with the improved clinical picture, as well as reduction in the length of ICU stay and hospital treatments. However, this case series was constrained by the limited sample size and the lack of comparator group of the enrolled patients.

The ET-AS regimen described in our study generally showed higher efficacy and acceptable tolerability for severe *P. falciparum* patients. Although our report was limited by the small number of patients and heterogeneous patient characteristics, these preliminary results suggest that ET-AS regimens could benefit patients with severe *P. falciparum* malaria.

### Electronic supplementary material

Below is the link to the electronic supplementary material.

Supplementary Material 1

Supplementary Material 2

## Data Availability

All data generated and/or analyzed are available from the corresponding author on reasonable request.

## Abbreviations

*P. Falciparum*: *Plasmodiun* falciparum
ET-AS: Exchange transfusion combined with a*rtesunate*
RCE: Red cell exchange
TPE: Therapeutic plasma exchange
TNF-α: Tumor necrosis factor
HB: Hemoglobin
HCT: Hhematocrit
PLT: Platelet count
LDH: Lactic dehydrogenase
Cr: Creatinine
AST: Aspartate transaminase
TB: Total bilirubin
ICU: Intensive care unit
GCS: Glasgow coma scale
AS: Artesunate
ACD-A: Acid citrate dextrose-A
