# Supplementary material for: Exchange transfusion combined with artesunate (ET-AS) as a safe and effective therapy in severe P. falciparum malaria: a case series
Source: BMC Infect Dis. 2024 Jun 19;24:603. doi: 10.1186/s12879-024-09381-2 (PMC11186206; doi:10.1186/s12879-024-09381-2)
Supplement: Supplementary file 2 — Supplementary Material 2 [file 12879_2024_9381_MOESM2_ESM.pdf]

SPRINGER NATURE ***Research Participant Publication Approval***

Consent for publication of material relating to the Research Participant in a Springer Nature product

This section to be completed by the Corresponding Author

Manuscript Number/ISBN (if applicable): a0bcf754-fe44-4ba1-89ba-26088f52deed

Journal/Book/Proceedings/Publication Name (if applicable): BMC Infectious Diseases (the "Publication")

Proposed Title of Article/Chapter/Paper/Product:

Exchange Transfusion combined with Artesunate (ET-AS) as a safe and effective Therapy in Severe *P. falciparum* Malaria: A case series (the "Contribution")

Format and description of material featuring the Research Participant:

This case series aims to describe the efficacy and safety of the exchange transfusion combined with artesunate regimen in severe P. falciparum malaria.All procedures in studies involving human participants were approved by the Ethics Committee of Jining Medical University， the research participant provided informed consent for their involvement in the study. The data for our case series was collected retrospectively from medical records of patients diagnosed with malaria at our institution between 2014 and 2023. The variables of interest, such as clinical characteristics, laboratory findings, thin or thick smears and treatment outcomes, were extracted from these medical records.

To ensure accuracy and consistency in data collection, a standardized data extraction form was used. Trained researchers carefully reviewed the medical records and recorded the relevant information. Any discrepancies or uncertainties were discussed and resolved through consensus among the research team.Numeric variables, such as age and laboratory values, were recorded as they appeared in the medical records. Categorical variables, such as gender and clinical outcomes, were calculated based on the available data in the records.all personal identifying information was treated confidentially and that appropriate measures were taken to protect the participant's privacy. We express gratitude to your participant for their contribution to the study.

(the "Material")

Corresponding Author's Name (the "Author"): Hao Zhang

Corresponding Author's Address: Affiliated Hospital of Jining Medical University, Jining 272029, Shandong Province, China.

Research Participant's Name: Shenhong Tan, Haiqiang Wang, Shui Dong, Yanqun Shi, Shui Wang, Hailong Luan,Weibo Yang, Cunmi Geng

Research Participant's Address: Jining 272029, Shangdong Province,China

SPRINGER NATURE ***Research Participant Publication Approval***

This section to be completed by the research participant or authorised representative:

I give my express consent for the Material, which may contain personal data including health data, to appear in the Contribution and be published by the Springer Nature Group, its licensees (the "**Publisher**") and/or other publishers in the Publication and associated works, in any form or medium as follows:

1. The Material may be published in the Contribution in print and/or electronic versions of the Publication. This means that the Publication may be published and distributed worldwide in print form and on the internet and in any other formats that may be used now and in the future, and may be accessible by the general public.
2. If the Contribution is published open access this means that there is **no restriction on access** to the Material and some restrictions on use of the Material by readers, dependent on the licence under which it is published. Please note that Springer Nature encourages use of the CC BY licence for maximum dissemination and reuse of open access material. Please see https://creativecommons.org/licenses/ for further information.
3. The Material may also be shared with companies affiliated with Publisher as well as with any third parties in order to be used in full or part in other publications, whether in print, online or other formats.
4. While the Research Participant's name will not be published, anonymity cannot be guaranteed.

I will not receive any payment or royalties in connection with the use and publication of the Material.

I warrant that I have the full right, power and authority to sign this consent form as the person (or on behalf of the person) shown or portrayed in the Material. I can withdraw my consent at any time without giving reasons. This does not affect the lawfulness of the data processing that has taken place before the withdrawal.

Signature of Research Participant: Shanhong Tan Haiqiang Wang Shui Dong Yan Qun Shi Hailong Luan Cunmi Geng weibo gong Date: 2023-11-08

If the Research Participant is co-signing with a parent, guardian or attorney:

Print Name: ____________________

Relationship to Research Participant: ____________________

Signature: ____________________ Date: ____________________

SPRINGER NATURE ***Research Participant Publication Approval***

Privacy Notice

By means of this document, the Author (as specified above) in their role as the data controller wants to inform you about the processing of your Personal Data in connection with publishing the Contribution and/or Publication. Below you will find information on what kind of Personal Data the Author collects from you, how they use it, for which purposes they use it, with whom they share it and which rights you have regarding the Processing of your Personal Data under the General Data Protection Regulation ("GDPR"). This privacy notice also provides information about the processing activities of the Publisher; information about the privacy practices of the Publisher can be found here https://link.springer.com/privacystatement and in the privacy policy of the Publication.

1. **Definitions**

Personal Data: means any information relating to a natural person who can be identified, directly or indirectly, in particular by reference to an identifier such as a name, an identification number, location data, or an online identifier.
Processing: means any operation which is performed on Personal Data, such as collection, recording, organising, structuring, storing, adaptation or any kind of disclosure or other use.

2. **Purposes of Processing**

In this section, you will be informed about the Personal Data the Author Processes, the purposes the Author uses the Personal Data for and the legal bases for the Processing of your Personal Data. Your Personal Data will be transferred to the Publisher (as specified above), where the Contribution and/or the Publication will be published (see 3 below). Thus, the Author also informs you about the Processing by such Publisher. Please find further information with regard to details of Publisher's Processing of Personal Data here: https://www.springernature.com/gp/legal/privacy-statement/11033522

2.1 **General Information concerning you (e.g. your name and address) will be Processed as far as they are necessary for publishing the Contribution and/or Publication. The legal basis for this Processing activity is the necessity for the performance of this Research Participant Publication Approval (Art. 6 (1) 1 lit. b GDPR). In case the Material contains personal health data, the legal basis for this Processing is your express consent (Art. 9 (2) lit. a GDPR).**

2.2 **Information relating to the film images/recordings/photographs of your likeness/voice and sound will be Processed as set out in the Research Participant Publication Approval. The legal basis for this processing activity is your consent (Art. 6 (1) 1 lit. a, 9 (2) lit. a GDPR).**

2.3 **Information concerning you or relating to the film images/recordings/photographs of your likeness/voice and sound will be Processed as far as the Processing is necessary for compliance with a legal obligation (e.g. tax law requirements). The legal basis for this Processing activity is Art. 6 (1) 1 lit. c GDPR.**

2.4 **Information concerning you or relating to the film images/recordings/photographs of your likeness/voice and sound will be Processed as far as the Processing is necessary for the establishment, exercise or defence of legal claims. The legal basis for this processing is the legitimate interest in being able to exercise the rights in the Contribution and/or Publication and defense against any legal claims in conjunction with the Contribution and/or Publication (Art. 6 (1) 1 lit. f GDPR). In case the Material in dispute contains personal health data, the legal basis for this processing is Art. 9 (2) lit. f GDPR.**

3. **Data Transfer and Disclosure**

Your Personal Data will be transferred to the Publisher of the Contribution/Publication, i.e. a publisher of the Springer Nature Group, their respective agents and licensees or other publishers . The legal basis for this Processing activity is your consent, Art. 6 (1) 1 lit. a, 9 (2) lit. a GDPR.
The Publisher might transfer the Personal Data to other third parties, e.g. service providers. Below is a list of functions and services which may require the use of third party service providers:

- Advertising services;
- Analytics services;
- Hosting and content delivery network services;
- IT services.

These service providers are only allowed to use the Personal Data shared with them for the specific task they have been hired to and for no other purposes. Any processing by and transfer to these service providers is based on Art. 28 GDPR in conjunction with a data processing agreement that is concluded with them.
In all of these cases, your Personal Data may be transferred outside of the country in which it is collected. Please note that other countries may not provide the same level of data protection as your country of origin. To ensure that the transfer does not disproportionately interfere with your rights and freedoms, the so-called EU Standard Contractual Clauses have been concluded with recipients outside the European Economic Area. The EU Standard Contractual Clauses can be viewed here: https://ec.europa.eu/info/law/law-topic/data-protection/data-transfers-outside-eu/model-contracts-transfer-personal-data-third-countries_en

4. **Data Retention**

Your Personal Data will be retained until you withdraw your consent, which is possible at any time without giving reasons.

5. **Data Subject Rights**

You may, depending on the specific circumstances, be entitled to exercise the following rights free of charge:

(a) require (i) information whether your Personal Data is retained and (ii) access to and/or (iii) duplicates of your Personal Data retained, including the purposes of the Processing, the categories of data concerned, and the recipients or categories of recipients to whom the data are disclosed and where possible, the envisaged period for which the personal data will be stored, or, if not possible, the criteria used to determine that period;

(b) request proper rectification, removal or restriction of your Personal Data if the processing does not comply with the provisions of applicable data protection law; in case your Personal Data is processed by third parties, the Author will forward your request for rectification, removal or restriction also to such third parties unless this proves impossible or involves disproportionate effort;

(c) withdraw your consent to processing of your Personal data at any time without impact to data Processing activities that have taken place before such withdrawal;

(d) receive your Personal Data in a structured, commonly used and machine-readable format and to transmit those data to another controller without hindrance from our side; where technically feasible you shall have the right to have the Personal Data transmitted directly from us, to another controller;

(e) **object at any time, out of legitimate and reasonable grounds relating to your particular case, that your Personal Data shall be subject to a processing;**

(f) **object to the use of your Personal Data for direct marketing at any time;**

(g) lodge complaints before the competent data protection regulators;

(h) require to not be subject to any automated decision making, including profiling (automatic decisions based on data processing by automatic means, for the purpose of assessing several personal aspects) which produce legal effects on you or affects you with similar significance.

In case you want to exercise your rights, please use the Author's contact data mentioned in the Research Participant Publication Approval.
